# Retraction of: Lithium treatment extends human lifespan: findings from the UK Biobank

**DOI:** 10.18632/aging.206118

**Published:** 2024-09-15

**Authors:** Elisa Araldi, Catherine R. Jutzeler, Michael Ristow

**Affiliations:** 1 Energy Metabolism Laboratory, Institute of Translational Medicine, Swiss Federal Institute of Technology (ETH) Zürich, Schwerzenbach CH-8603, Switzerland; 2 Center for Thrombosis and Hemostasis and Preventive Cardiology and Preventive Medicine, Center for Cardiology, University Medical Center of the Johannes Gutenberg University Mainz, Mainz D-55131, Germany; 3 Biomedical Data Science Lab, Institute of Translational Medicine, Swiss Federal Institute of Technology (ETH) Zürich, Zürich CH-8008, Switzerland; 4 Institute of Experimental Endocrinology and Diabetology, Charité Universitätsmedizin Berlin, Berlin D-10117, Germany

**Keywords:** geroprotection, human, nutrition, pharmacology, psychiatry

**This article has been retracted:** The Authors asked about the retraction because they received a note from the UK Biobank: “The UK Biobank Research Development Team has informed us that the use of a subset of prescription data in our analyses does not comply with the Control of Patient Information (COPI, 2020) notice. Specifically, ‘these prescription data could only be used for the purpose of COVID-19 research’. While we have analyzed the impact of these prescription drugs on COVID-19 mortality, as contained in the present manuscript, any additional analyses, as also contained within, appear to be incongruent with the COPI notice. We hence abide by the request of UK Biobank Research Development Team to withdraw the entire publication. We apologize for any inconvenience this may cause. It should be noted that the scientific content, methods and validity of the results in the entire publication remain unaffected by both the request and the subsequent retraction.” All authors accepted to retract the manuscript.

